# Natural Genetic Resources from Diverse Plants to Improve Abiotic Stress Tolerance in Plants

**DOI:** 10.3390/ijms21228567

**Published:** 2020-11-13

**Authors:** Seher Yolcu, Hemasundar Alavilli, Byeong-ha Lee

**Affiliations:** 1 Department of Life Science, Sogang University, Seoul 04107, Korea; seher808@gmail.com; 2 Department of Biochemistry and Molecular Biology, College of Medicine, Korea University, Seoul 02841, Korea; alavilli.sundar@gmail.com

**Keywords:** genetic diversity, stress tolerance, natural variations, natural accessions, extremophiles, crop progenitors, CRISPR

## Abstract

The current agricultural system is biased for the yield increase at the cost of biodiversity. However, due to the loss of precious genetic diversity during domestication and artificial selection, modern cultivars have lost the adaptability to cope with unfavorable environments. There are many reports on variations such as single nucleotide polymorphisms (SNPs) and indels in the stress-tolerant gene alleles that are associated with higher stress tolerance in wild progenitors, natural accessions, and extremophiles in comparison with domesticated crops or model plants. Therefore, to gain a better understanding of stress-tolerant traits in naturally stress-resistant plants, more comparative studies between the modern crops/model plants and crop progenitors/natural accessions/extremophiles are required. In this review, we discussed and summarized recent progress on natural variations associated with enhanced abiotic stress tolerance in various plants. By applying the recent biotechniques such as the CRISPR/Cas9 gene editing tool, natural genetic resources (i.e., stress-tolerant gene alleles) from diverse plants could be introduced to the modern crop in a non-genetically modified way to improve stress-tolerant traits.

## 1. Introduction

Since the birth of *Agrobacterium tumefaciens*-mediated genetic transformation in plant biotechnology [1], many genes have been identified and used to improve crop traits—notably, stress tolerance, which is the main topic of this review. Some genes have been successful, while some have not [2]. However, even a gene successful in one particular crop does not always work for another crop. This indicates the presence of diverse genetic functions and regulation systems in plants. Thus, one approach to overcome this hurdle of crop improvement could be to isolate the diverse alleles from various natural resources.

In the course of the domestication and continuous artificial selection, genetic diversity in the wild cultivars has been lost in many modern crop cultivars [3,4,5]. Crop breeding favored yield over other traits such as stress tolerance, which could be replaced by farmers’ care and agricultural techniques [2,6]. Thus, compared to the modern crop varieties, wild ancestors of crops could effectively tolerate and thrive under a wide range of weather fluctuations and unfavorable habitats [2,5,7,8]. Hence, the wild accessions in many crop plants can be considered primary reservoirs of genetic diversity and deserve much more attention to be explored for their stress-tolerant traits.

In addition to the genetic resources from wild accessions, genes from diverse plants such as extremophiles, which are accustomed to extreme environmental conditions, can also serve as treasure troves for stress-tolerant alleles [3,9,10,11,12,13,14].

In this review, we look into the current studies on stress-tolerant gene alleles from various germplasms that render advantageous traits in stress tolerance and provide an opportunity to think about possible applications. In Table 1, we also summarize the stress-tolerant alleles from diverse resources that show superior functions in stress tolerance.

## 2. Loss of Stress Tolerance during Domestication

### 2.1. Limited Genetic Diversity in Domesticated Crops

Crop domestication through artificial selection often results in opposition to natural selection [40]. For example, farmers favored the trait of non-shattering seeds, which is obviously an unfavored trait for plants in nature that need to spread their offspring widely [41]. The breeding programs mostly aim to develop high yielding crop varieties [2]. Thus, domestication of crops has resulted in increased productivity but limited genetic diversity, often by losing useful alleles such as stress-tolerant genes [42]. During domestication processes, beneficial traits can be lost by gene loss, changes in gene regulation, and gene activity modification (i.e., sequence variations in coding sequence).

### 2.2. Gene Loss

Munns et al. examined natural diversity in shoot Na^+^ exclusion within ancestral wheat germplasms. An ancestor of modern wheat, *Triticum monococcum* carries *Nax1* and *Nax2* loci while modern durum wheat (*Triticum turgidum*) or bread wheat (*Triticum aestivum*) does not [31,43]. The introduction of the *T. monococcum Nax2* locus encoding *TmHKT1;5-A* gene to durum wheat by genetic crosses or molecular transformation dramatically decreased Na^+^ accumulation in the leaves and increased grain yield on saline soils [31]. *High-affinity K^+^ Transporter* (*HKT*) is one of the genes that is found only in plants, facilitating mostly monovalent cation (i.e., K^+^ and Na^+^) transport and playing important roles in the regulation of K^+^ and Na^+^ transport, and hence the salt tolerance in plants [44,45,46,47]. Thus, this gives an example that a gene responsible for stress tolerance in a wheat ancestor was lost during domestication to make durum wheat and bread wheat.

### 2.3. Alterations in Gene Expression

In addition to stress-tolerant gene loss, modifications of gene expression can also occur during domestication by changing the promoter activity. Bian et al. uncovered that the gene expression levels of class-B type heat shock factor, *HSFB2b*, are correlated with the degrees of salt stress tolerance in soybean (*Glycine max*) [32]. *HSFB2b* appeared to promote flavonoid accumulation by activating the expression of flavonoid biosynthetic genes and relieving the inhibition of flavonoid biosynthesis, which led to salt stress tolerance. Among the four haplotypes of the *HSFB2b* promoters in the analyzed soybeans, ones from salt-tolerant wild accessions displayed higher *HSFB2b* promoter activity than the domesticated soybean cultivars. This suggests that the strong selection was imposed on the promoter regions of *HSFB2b* during domestication and that the wild accessions could provide a strong allele to combat against salt stress [32]. In addition, many genome-wide association studies (GWAS) in maize have also revealed the promoter sequences as a genic area of the domestication target (see below).

### 2.4. Variations in Coding Sequence

In addition, natural variations in the open reading frame could also lead to a loss of beneficial traits during crop domestication. After analyzing the differential patterns of heat stress acclimation in the course of domestication between wild tomato (*Solanum pennellii*) and cultivated tomato (*Solanum lycopersicum*), Hu et al. found an alternative splicing variation in the *HsfA2* gene which encodes a major heat inducible transcription factor [5]. The natural variation in wild species caused alternative splicing of the *HsfA2* gene, resulting in the synthesis of a HsfA2-II isoform for rapid heat stress acclimation in wild species compared to the cultivated tomato [5].

In another report, Wang et al. compared the Na^+^/K^+^ ratios in roots of salt-sensitive and -tolerant tomato cultivars under salt stress and processed the results through GWAS analysis [4]. They found that the gene *SlHAK20* was associated with salt stress tolerance: The natural variations in the *SlHAK20* coding sequence appeared to be the reason for the different levels of salt tolerance in tomato accessions. The *SlHAK20* encoded a member of the clade IV High-Affinity K^+^/K^+^ Uptake Permease/K^+^ Transporter (HAK/KUP/KT) transporters and functioned in the transport of Na^+^ and K^+^. Salt-tolerant wild tomato accessions contained a “strong” *SlHAK20* allele (haplotype 1) which efficiently lowered the Na^+^/K^+^ ratio in plants and brought about enhanced salt tolerance. The activity of domesticated tomato *SlHAK20* (haplotype 2) was low, indicating the coding sequence variations during domestication caused the loss of SlHAK20 activity, hence the loss of salt tolerance [4].

## 3. Alleles for Stress Tolerance

### 3.1. Arabidopsis

*Arabidopsis thaliana* accessions (ecotypes) are broadly distributed to the various regions of the world, and this distribution contributes to the genetic variations of *Arabidopsis* that are crucial for stress adaptation [48]. Thus, genetic variations among the *Arabidopsis* accessions provide a valuable genetic model to find out both unique stress tolerance mechanisms and important stress-tolerant alleles [49]. For example, Jha et al. found a positive correlation between *AtAVP1* transcript levels and salinity tolerance [50]. In comparison to Col and C24, the Ler and Ws accessions displayed higher transcript abundance of *AtAVP1* in association with higher salt stress tolerance [50].

#### 3.1.1. RAS1

Shakdara (Shahdara, Sha) is an *Arabidopsis* accession from the Shakdara Valley of Tadjikistan, which is geographically covered with mountainous landscapes with low precipitation [51]. Because of its origins, the Sha ecotype typically displays tolerance to multiple abiotic stresses [15,51,52,53,54]. Ren et al. [15] reported a gene, *Response to ABA and Salt 1* (*RAS1*), which encodes a transcription factor-like protein. The *RAS1* allele from the salt-sensitive accession Ler encodes a 230 amino acid-length protein, while the *RAS1* allele in Sha only produces a truncated polypeptide with 209 amino acid residues due to the premature stop codon [15]. RNAi-caused *RAS1* reduction in Ler reduced salt sensitivity, and overexpression of the Ler *RAS1* allele increased salt sensitivity, supporting the correlation of the *RAS1* coding sequence variations with salt sensitivity. The *RAS1* gene acts as a negative regulator of salt tolerance; hence, the functional loss of *RAS1* gene could render the salt tolerance. Thus, it is expected that the allele modification resulting in the C-terminal truncation and loss of its activity enabled Sha to become salt tolerant in the course of its evolution [15].

#### 3.1.2. LRR-KISS

A genome-wide association study (GWAS) with 160 accessions of *Arabidopsis* identified one single nucleotide polymorphism (SNP) associated with natural variations in dry weight at 500 mM NaCl. The SNP was located in the promoter region of the *Leucine-Rich Repeat Kinase Family Protein Induced By Salt Stress* (*LRR-KISS*) gene [16]. Salt stress did not affect the rosette size in *Arabidopsis* accession with high *LRR-KISS* expression (Cen-0), while the same stress greatly reduced relative rosette size in the accession with low *LRR-KISS* expression (HR-5). This suggests that the natural variations in the promoter activity are associated with salt stress tolerance—the higher the *LRR-KISS* transcript levels, the larger the rosettes under salt stress.

### 3.2. Rice

Rice is originally a chilling-sensitive crop derived from tropical or sub-tropical regions of Asia. Rice cultivars include two subspecies, *indica* and *japonica* [55,56]. Compared to *indica*, the *japonica* rice cultivars were found to be more cold-tolerant and grow at higher altitudes and latitudes and temperate zones [11,17,57].

#### 3.2.1. Cold-Tolerant Alleles from *Japonica* Cultivar

To understand the genetic basis of enhanced cold tolerance in *japonica*, several genes with natural variations were identified and characterized [11,13,17,18]. Some of the superior alleles from *japonica* in cold tolerance are described below. Some that were not described due to the space limits are included in Table 1.

##### COLD1

The *CHILLING-TOLERANCE DIVERGENCE 1* (*COLD1*) gene encodes a plasma membrane and endoplasmic reticulum localized regulator of G-protein which activates Ca^2+^ influx upon cold sensing [17]. *COLD1* was identified through a quantitative trait locus (QTL) analysis in recombinant inbred lines from a cross between chilling-tolerant *japonica* and chilling-sensitive *indica*. *COLD1* allele from *japonica* and *indica* contained several SNPs including an SNP in the fourth exon (SNP2), which was shown to be associated with chilling tolerance. An expanded comparative sequence analysis of *japonica* cultivars, *indica* cultivars, and wild rice revealed polymorphism of T/C in chilling-sensitive rice (*indica*) and A in chilling-tolerant rice (*japonica* and wild rice *Oryza rufipogon*) at SNP2, which resulted in Met187/Thr187 in chilling-sensitive rice and Lys187 in chilling-tolerant rice [17]. Thus, it is likely that chilling-tolerant *COLD1* allele from *O. rufipogon* was selected during *japonica* domestication to confer the chilling tolerance trait in *japonica* [17].

##### HAN1

Similarly, a recent report demonstrated the *HAN1* gene contribution to chilling tolerance in temperate *japonica*. The *HAN1* gene encodes an oxidase that converts the active jasmonoyl-L-isoleucine (JA-Ile) to an inactive form, 12-hydroxy-JA-Ile (12OH-JA-Ile), and it further modulates the jasmonic acid-mediated chilling responses [13]. Overexpression and mutation analysis of *HAN1* suggested that *HAN1* negatively regulates chilling tolerance. While both coding sequences of *HAN1* alleles from *indica* cultivar (Teqing) and *japonica* cultivar (02428) showed the same functional and physiological activities, the allelic variation for chilling tolerance variations was found in the *HAN1* promoter [13]. The promoter analysis in 101 rice accessions identified an SNP in MYB cis-elements at the promoter of *HAN1* in most chilling-tolerant *japonica* cultivars, which might be responsible for the adaptation of *japonica* cultivars to temperate climates, probably by reducing the *HAN1* expression.

##### CTB4a

QTL analysis on recombinant inbred lines from a cross between cold-sensitive Towada and the cold-tolerant *japonica* landrace KUNMINGXIAOBAIGU (KMXBG) at booting stage identified the *cold tolerance at booting stage 4a* (*CTB4a*) encoding a leucine-rich repeat receptor-like kinase [11]. Despite the multiple SNPs and indels both in the promoter regions and the coding regions of the *CTB4a* alleles from KMXBG and Towada, a strong correlation was found between the *CTB4a* expression levels and cold tolerance, suggesting that the nucleotide polymorphisms in the promoter regions account for different cold sensitivity. Further analysis revealed that upregulation of *CTB4a* is positively correlated with ATP synthase activity, ATP contents, seed setting, and yield under cold stress [11].

##### bZIP73

In rice, there are 89 members of the basic leucine zipper (bZIP) family proteins and some of bZIP proteins have been identified to function in cold stress responses in rice [58,59]. Using association analysis and population genetics of *bZIP* genes combined with expression profiles under stress, *bZIP73* was identified to be associated with low temperature seedling survivability [18]. The *bZIP73* contained a single SNP in the coding sequence; at 511 bp, G in *japonica* and A in *indica*, resulting in glutamate in *japonica* and lysine in *indica* at the one hundred and seventy-first amino acid. This single SNP in *bZIP73* was responsible for differences in cold tolerance between *japonica* and *indica* as evidenced by overexpression of each allele and the introgression of the *indica bZIP73* allele to *japonica*. The *japonica bZIP73* allele, working with its interacting partner *bZIP71*, is a superior allele in comparison to the *indica* allele to facilitate cold adaptation during *japonica* domestication [18].

#### 3.2.2. Heat- and Drought-Tolerant Alleles from Indica Cultivar

Unlike *japonica* cultivars, *indica* cultivars predominantly grow in tropical and subtropical regions [18,60]. Wild progenitors from a low moisture region such as *O. barthii, O. australiensis, O. glaberrima*, *O. longistaminata*, and *O. punctata* could be able to provide drought- or heat-tolerant candidate genes to cultivated rice [61].

##### TT1

CG14 line originated from an African wild rice cultivar *O. glaberrima* is more tolerant to heat stress than Wuyunjing, an Asian cultivar. Through map-based cloning, Li et al. identified the causal gene at the *Os03g0387100* locus and named it as *Thermo-tolerance 1* (*TT1*) [25]. Multiple sequence alignment of the *TT1* locus with other species revealed the existence of three SNP variations in the *Oryza glaberrima TT1* (*OgTT1*) allele compared to its counterpart *Oryza sativa TT1* (*OsTT1*). One of the variations in the *OgTT1* coding sequence caused the substitution of histidine instead of arginine at the ninety-ninth position in the protein sequence of *OsTT1* from Wuyunjing [25]. The *OgTT1* overexpressing lines displayed predominantly higher thermal tolerance than the *OsTT1* overexpressing lines. This suggests that the *OgTT1* is a superior allele to *OsTT1* in rendering heat tolerance [25]. *TT1* encodes an α2 subunit of the 26S proteasome [25].

##### OsDREB1F

In rice, *dehydration-responsive element binding 1F* (*DREB1F*) gene is known as one of the key drought stress-responsive transcriptional activator genes. To assess the allelic variations and molecular evolution of *DREB1F*, *DREB1F* alleles from *indica* wild rice germplasms and various accessions were compared along with the *Oryza sativa DREB1F* (*OsDREB1F*) [26]. The haplotype analysis classified the *DREB1F* alleles into 8 haplogroups and the alleles from drought-tolerant lines fell into H5 haplogroup with C changed to G at the three hundred and sixty-third nucleotide from the translation start site, resulting in a substitution of glutamate in the place of aspartate. This natural variation is associated with drought tolerance in the H5 haplogroup compared to the other variants [26].

#### 3.2.3. Drought- and Flooding-Tolerant Alleles from Upland Rice and Lowland Rice

Two major rice ecotypes include upland rice and lowland rice [62]. While lowland rice grows on flooded paddies, upland rice grows on dry soil; hence, upland rice cultivars are drought tolerant.

##### OsLG3 and OsCBL10

Gene association analysis revealed that the natural variation in the *OsLG3* promoter is associated with osmotic tolerance [12]. Upon drought stress, *OsLG3*, an ethylene response factor (ERF) family transcription factor is more highly induced in IRAT109, a *japonica* upland rice cultivar, than in Nipponbare, a *japonica* lowland rice cultivar. Introduction of the *OsLG3* allele from IRAT109 to rice improved drought tolerance, attributing the IRAT109 variation in the *OsLG3* promoter to enhanced drought tolerance [12]. Higher expression of *OsLG3* in IRAT109 because of variations in the promoter efficiently induced the transcription of downstream stress-responsive genes and the reactive oxygen species (ROS) scavenging genes under drought, causing increased drought tolerance. Similarly, natural variations in the *OsCBL10* promoters of upland and lowland rice are shown to be associated with flooding tolerance [27]. *Japonica* lowland rice variations in the *OsCBL10* promoter suppress the *OsCBL10* expression in response to flooding, affecting downstream Ca^2+^ mediated signaling toward flooding tolerance in seed germination [27]. These results again recapitulate that the regulation on the promoter activity is one of the selection targets in rice domestication.

### 3.3. Maize

#### 3.3.1. ZmDREB2.7

In maize, drought-responsive *ZmDREB2.7* displays natural variations in association with drought tolerance. DNA polymorphisms at the 5′ untranslated regions of *ZmDREB2.7* gene were shown to be important in determining the degrees of drought tolerance in maize varieties. Liu et al. found that the *ZmDREB2.7* allele from drought-tolerant tropical or subtropical varieties (TST; CIMBL70, 91, 92 and CML118) was rapidly induced by moderate drought in comparison with the drought-sensitive variety (Shen5003) [28]. In return, the TST *ZmDREB2.7* allele was more effective in inducing drought stress tolerance. These data suggest that the tolerant *ZmDREB2.7* allele is a beneficial genetic resource for drought-tolerant crop development.

#### 3.3.2. ZmVPP1

In maize, the *ZmVPP1* encodes for a vacuolar-type H^+^ pyrophosphatase. A GWAS study of drought tolerance at the maize seedling stage identified 83 genetic variants and uncovered that two variants (indels at -397 (366 bp) and -126 (5 bp) upstream from the translation start site) at the promoter region of *ZmVPP1* are significantly associated with drought tolerance [3]. In particular, the 366-bp insertion of *ZmVPP1* alleles from drought-tolerant tropical/subtropical cultivars contained three MYB binding cis elements and was important in drought stress-responsive gene induction, hence the enhanced drought tolerance [3].

#### 3.3.3. ZmNAC111

Another GWAS study with 368 inbred lines identified the *ZmNAC111* gene associated with drought tolerance at the seedling stage [29]. Among the natural variations in the *ZmNAC111* gene, the 82-bp insertion of a miniature inverted-repeat transposable element (MITE) at the promoter was found to be most significantly associated with drought tolerance. The MITE was present in the *ZmNAC111* alleles from drought-sensitive B73 and Mo17 inbreds while it was missing in drought-tolerant CIMBL55, 70, 90, and CML118. The MITE insertion in the drought-sensitive *ZmNAC111* allele caused a reduction in the *ZmNAC111* expression in drought-sensitive cultivars, resulting in lower drought tolerance compared to drought-tolerant cultivars [29].

#### 3.3.4. ZmTIP1

Through a GWAS analysis, Zhang et al. recently established a significant association between the natural variations of *ZmTIP1* and drought tolerance [30]. *ZmTIP1* encodes an S-acyl transferase and its expression levels are correlated with the degrees of drought tolerance [30]. The *ZmTIP1* alleles from drought-tolerant cultivars contained a higher number of transcription factor binding elements in the promoter region than the alleles from drought-sensitive cultivars. These natural variations are responsible for higher *ZmTIP1* expression in drought-tolerant cultivars than drought-sensitive lines. However, the natural variations in the *ZmTIP1* coding region did not contribute to the levels of drought tolerance. ZmTIP1 may function through S-acylation of a calcium-dependent protein kinase (ZmCPK9) that facilitates the ZmCPK4 association with the plasma membrane.

### 3.4. Extremophiles

#### 3.4.1. MBF1c from a Polar Moss

*Polytrichastrum alpinum* is one type of polar moss that survives extreme conditions in the Antarctic. From this extremophile, a stress-responsive transcriptional coactivator gene, *multi-protein bridging factor1c* (*PaMBF1c*), was isolated and compared with *Arabidopsis MBF1c* (*AtMBF1c*) [14]. In previous work, the *AtMBF1c* gene was already identified as a key regulator for thermotolerance responses and the *AtMBF1c* gene overexpression enhanced heat tolerance in *Arabidopsis* [63]. When *Arabidopsis* transgenic plants overexpressing the *PaMBF1c* gene were compared with *AtMBF1c* overexpressing *Arabidopsis* lines, both lines showed high tolerance to heat stress [14]. Interestingly, the *PaMBF1c* overexpressing lines conferred enhanced tolerance to salt (NaCl) and ionic (LiCl) stresses at both germination and seedling stages in comparison to the WT and *AtMBF1c* overexpressing lines. In other words, *PaMBF1c* overexpression brought about tolerance to multiple stresses including heat, salt, and ionic stresses, which was not observed in the *AtMBF1c* overexpressing *Arabidopsis*. It is assumed that *PaMBF1c* might have evolved to adapt *P. alpinum* to the high salt conditions of the Antarctic, probably by optimizing amino acid residues for the multifunctional activity. PaMBF1c contains 14 amino acid variations different from *Triticum aestivum* MBF1c and AtMBF1c, the functions of which were shown to be involved in abiotic stress tolerance [64,65]. In conclusion, the data suggest that extremophiles could contain a more “useful” allele for improvement of abiotic stress tolerance in crops.

#### 3.4.2. Galactinol Synthase 1 from a Desert Shrub

*Ammopiptanthus nanus* is a desert shrub that can survive a wide temperature range, from −30 to 47 °C [66,67]. *Galactinol synthase1 (GolS1)*, a galactinol biosynthesis gene associated with stress tolerance, was shown to be cold-inducible in *A. nanus* [68,69]. Overexpression of *A. nanus GolS1* (*AnGolS1*) in tomato caused an increase of galactinol contents in young leaves under cold stress, activation of ethylene signaling, and induction of ethylene response factors (ERFs), which led to cold tolerance [33]. Among four tomato *GolS*s (*SlGolS1*, *SlGolS2*, *SlGolS3*, and *SlGolS4*) homologous to *AnGolS1*, only *SlGolS2* was cold-inducible, but at low levels, and believed to be a functional homolog of *AnGolS1*. AnGolS1 showed higher catalytic activity than SlGolS2 [33]. Thus, it is assumable that these features of *AnGolS1* such as a higher inducibility and a higher catalytic activity might confer higher cold stress tolerance than *SlGolS2*.

#### 3.4.3. Sodium Proton Antiporters from Halophytes

Overexpression of the halophyte *Suaeda salsa NHX1* (*SsNHX1*) increased cold and salt tolerance in *Arabidopsis* [34]. Yadav et al. also reported that transgenic tobacco plants overexpressing the *SbSOS1* gene from an extreme halophyte *Salicornia brachiata* showed higher salt tolerance compared to the WT plants [35]. Although these results were already observed in *AtNHX1* or *AtSOS1* overexpressing *Arabidopsis* [70,71], the halophyte *SsNHX1* gene overexpressing plants grew normally even after 200 mM NaCl treatment [34]. In contrast, *AtNHX1* or *AtSOS1* overexpressors showed a limited salt tolerance only up to 200 mM NaCl and could not survive at NaCl concentrations higher than 200 mM [72]. Thus, it is likely that these halophyte genes function better under stress than the glycophyte homologs.

#### 3.4.4. HKT1;1 from Halophytes

High-affinity K^+^ transporter, HKT1;1 (previously known as HKT1), plays an important role in reducing the phytotoxicity of Na^+^ by controlling shoot Na^+^ levels and maintaining the balance between Na^+^ and K^+^ ions under salt stress. Originated from the Namibian desert in Southern Africa, *Mesembryanthemum crystallinum* (also known as ice plant) is one of the extremophiles and is extremely salt stress tolerant, withstanding up to 0.5 M NaCl [73]. Arabidopsis *HKT1;1* homolog gene, *McMKT2* (now renamed *McMKT1;2*) was isolated from *M. crystallinum* and found to have several distinct features compared to *AtHKT1;1* [36]. For instance, the McHKT2 amino acid sequence contains 13 contiguous threonine residues between the first and second transmembrane pore domains as compared to the amino acid sequences of HKT1 from other plant species [36]. *AtHKT1*-overexpressing Arabidopsis is known to display salt-sensitive phenotypes [74,75]. However, *McMKT2* overexpressing Arabidopsis showed significantly high salt tolerance. Thus, it is speculated that the natural variations in *McMKT2* from halophyte *M. crystallinum* are responsible for these functional differences when overexpressed. Indeed, *HKT1* orthologs with natural variations from soybean and maize also brought about enhanced salt tolerance in tobacco plants when overexpressed [76,77,78].

Recent studies determined amino acid variations responsible for the *HKT1* functional differences between glycophyte and halophyte. *AtHKT1;1* from the glycophyte *Arabidopsis* and *TsHKT1;2* from the halophyte *Thellungiella salsuginea*, a model extremophile plant, share high DNA and protein sequence identities. However, they have different features in terms of ion selectivity and salt stress responses [37,79]. When the two genes were expressed in the *Arabidopsis hkt1-1* mutant under control of the *AtHKT1;1* promoter, *TsHKT1;2* expressing lines showed higher tolerance to salinity, a higher fresh biomass, and a lower shoots/roots Na^+^ ratio than wild-type or *AtHKT1;1* expressing lines, exemplifying that the *HKT1* allele from an extremophile *(TsHKT1;2)* is a superior allele in handling salt stress [37,38,79]. By comparative analysis and protein modeling, an amino acid residue important for each activity was found in the second pore loop domain; the two hundred and seventh aspartate residue in TsHKT1;2 and the two hundred and eleventh asparagine residue in AtHKT1;1. When *TsHKT1;2*^D207N^ (TsHKT1;2 with Asp at the two hundred and seventh amino acid switched to Asn) was expressed in the K^+^ transporter-deficient yeast, *TsHKT1;2^D207N^* expression resulted in lower salt tolerance than its original *TsHKT1;2* expression, but slightly higher than *AtHKT1;1* expression. These results indicate the superior function of the *TsHKT1*;1 allele associated with the amino acid variation [37,38]. A similar functional determinant at the amino acid variation in the second pore loop was identified in another extremophile *Eutrema parvula HKT1;2*, indicating that the presence of Asn or Asp in the second pore loop determines the cation selectivity in the HKT1 transporters [74]. This finding also fortifies the notion that the extremophiles could contain the functionally superior alleles.

More importantly, *AtHKT1;1^N211D^* (AtHKT1;1 with Asn at the two hundred and eleventh amino acid changed to Asp) expression in the *Arabidopsis hkt1-1* mutant showed significantly enhanced growth in comparison to the original *AtHKT1;1* expressing *hkt1-1* lines [38]. Using this information, Vu et al. applied the CRISPR/Cpf1-mediated homologous recombination technique to replace the original Asn at the two hundred and seventeenth amino acid of the tomato *HKT1;2* allele with Asp residue important for the activity of the superior *TsHKT1;2* allele in salt stress [39]. This gene edited *SlHKT1;2^N217D^* tomato showed enhanced salt tolerance at the germination stage under 100 mM NaCl and the edited allele was successfully inherited to the next generation [39].

## 4. Concluding Remarks

Conventionally, most of the physiological characterization studies, thus far, aimed to elucidate a gene function by relying on genes from model plants or modern cultivars through either mutant or overexpression-based approaches. Recently, many researchers reported the utilization of the genetic resources from wild crop progenitors and extremophiles and proved many of them to be better alleles than alleles from model plants in crop improvement against abiotic stresses. Therefore, it would be needed to expand our efforts to understand the natural genetic variations in the wild progenitors or accessions which have been adapted to extreme environments. Understanding of their stress-evading strategies will enable us to plan and design the breeding and biotechnological programs to generate crop plants with multifaceted stress tolerance [80,81].

The recent bioinformatic technical advancements including whole genome surveys, gene targeted surveys, and genome-wide association studies successfully demonstrated and uncovered the complex causative elements with very high precision in various plant species. They could be highly instrumental to expand our knowledge on the genetic information of untapped stress-tolerant wild ancestors and to learn the selection impact over wild cultivar germplasm during domestication. As we summarized, many natural variations including indels in the promoters and SNPs in the coding sequences have been reported to be determinant factors of the levels of gene activity. To make this genetic resource more useful, it is necessary to carry out comparative studies among the alleles. Thus far, there are not many comparative studies of alleles from the extremophiles and temperate plants.

When combined with genetic resources and natural variations, the revolutionary CRISPR gene editing tool will facilitate crop improvement. For example, the negative regulators of stress tolerance could be deleted by the CRISPR gene editing tool to introduce the stress tolerance in the crop if the crop genome contains the negative regulator gene. In addition, the variations in the promoters and the coding sequences could be edited to be a superior allele. The CRISPR editing variants such as the base editors and the prime editors will be a tool of choice for introducing the favorable natural variations into the target genes. The above-described tomato *HKT1* editing study is one example of CRISPR application in crop improvement [39]. One thing to note is that the natural genetic resources might have not been selected during domestication due to its deleterious effects on yield. Thus, detailed characterization of the “superior” alleles must be performed to avoid any yield penalty in crop improvement.

## Figures and Tables

**Table 1:** Superior alleles from various plants in stress tolerance comparison studies.

Gene Linked with Abiotic Stress	Stress-Tolerant Plant Species/ Accessions	Stress-Sensitive Plant Species/ Accessions	Type of Abiotic Stress	Variations	Reference
*RAS1* Transcription factor-like protein	*Arabidopsis* accession *Shakdara (Sha)*	*Arabidopsis* accession Ler	Salinity	SNP in coding sequence resulting in a truncated polypeptide	[15]
*LRR-KISS* Leucine-rich repeat receptor kinase	*Arabidopsis* population Cen*-0*	*Arabidopsis* population HR*-5*	Salinity	SNP in the promoter	[16]
*COLD1* Regulator of G-protein signaling	*Oryza rufipogon* *Oryza sativa* subsp. *japonica*	*Oryza sativa* subsp. *indica*	Cold	SNP in the coding sequence resulting in Met187/Thr187 in indica and Lys187 in *japonica* and wild rice	[17]
*HAN1* JA-Ile hydroxylating oxidase	*Oryza sativa* subsp. *japonica*	*Oryza sativa* subsp. *indica*	Cold	SNP in MYB cis element at the promoter	[13]
*CTB4a* Leucine-rich repeat receptor-like kinase	*Oryza sativa* subsp. *japonica,* Kunmingxiaobaigu	*Oryza sativa* subsp. *japonica,* Towada	Cold	Nucleotide polymorphisms in the promoter	[11]
*bZIP73* Basic leucine zipper protein	*Oryza sativa* subsp. *japonica*	*Oryza sativa* subsp. *indica*	Cold	SNP in the coding sequence resulting in an amino acid change	[18]
*LTG1* Casein kinase	*Oryza sativa* subsp. *japonica*	*Oryza sativa* subsp. *indica*	Cold	SNP in the coding sequence resulting in an amino acid change	[19]
*qLTG3-1* Unknown protein	*Oryza sativa* subsp. *japonica* Italica Livorno	*Oryza sativa* subsp. *japonica* Hayamasari	Cold	71 bp deletion at the qLTG3-1 locus resulted in a frame shift in the sensitive cultivar	[20]
*Ctb1* Kelch repeat F-box protein	*Oryza sativa* subsp. *japonica* Norin-PL8	*Oryza sativa* subsp. *japonica* Kirara397	Cold	Several SNP in the promoter	[21]
*LTT7* Unknown protein	*Oryza sativa* subsp. *japonica* IL112	*Oryza sativa* subsp. *indica* GC2	Cold	Possible differences in cold stress-related cis-elements in the promoter	[22]
*qCTS-9* Unknown protein	*Oryza sativa* subsp. *japonica* Lijiangxintuanheigu	*Oryza sativa* subsp. *indica* Shanhuangzhan2	Cold	5 SNPs and one indel in the promoter	[23]
*qPSR10* Pectin lyase fold family protein	*Oryza sativa* subsp. *japonica*	*Oryza sativa* subsp. *indica*	Cold	SNP in the coding sequence resulting in an amino acid change	[24]
*OgTT1* Proteasome α2 subunit	*Oryza glaberrima* CG14	*Oryza sativa* Wuyunjing	Heat	SNP in the coding sequence resulting in amino acid change	[25]
*DREB1F* DRE binding transcription factor	Drought-tolerant *Oryza nivara, O. rufipogon, O. sativa* germplasm and accessions	Drought-sensitive *Oryza nivara, O. rufipogon, O. sativa* germplasm and accessions	Drought	Three SNPs in the coding sequence	[26]
*OsLG3* ERF family transcription factor	*Oryza sativa* subsp. *japonica* upland rice IRAT109	*Oryza sativa* subsp. *japonica* lowland rice Nipponbare	Drought	Three SNPs in the promoter	[12]
*OsCBL10* Calcineurin B-like protein	*Oryza sativa* subsp. *japonica* lowland rice Low88	*Oryza sativa* subsp. *indica* upland rice Up221	Flooding	Variations in the promoter	[27]
*ZmDREB2.7* DRE binding transcription factor	*Zea mays* tropical or subtropical varieties (TST) CIMBL70*,* 91*,* 92 and CML118	*Zea mays* Shen5003	Drought	DNA polymorphisms at the 5′ untranslated region	[28]
*ZmVPP1* Vacuolar-type H^+^ pyrophosphatase	*Zea mays* CIMBL55	*Zea mays* B73	Drought	DNA polymorphisms in the promoter	[3]
*ZmNAC111* NAC type transcription factor	*Zea mays* CIMBL55*,* 70*,* 92*,* and CML118	*Zea mays* B73 and Mo17	Drought	A MITE insertionin the promoter	[29]
*ZmTIP1* S-acyl transferase	*Zea mays* CIMBL55	*Zea mays* B73 and Mo17	Drought	Variations of transcription factor binding elements in the promoter	[30]
*Nax2* HKT1;5-A High-affinity K^+^ transporter	*Triticum monococcum*	*Triticum turgidum* and*Triticum aestivum*	Salinity	Gene loss in *T. turgidum* and *T. aestivum*	[31]
*HSFB2b* Class-B type heat shock factor	*Glycine soja* *Y20* and *Y50*	*Glycine soja* *Y0532*	Salinity	18 SNPs and/or indels in the promoter	[32]
*HsfA2* Heat inducible transcription factor	*Solanum pennellii*	*Solanum lycopersicum*	Heat	SNPs in intron 2 resulting in alternative splicing	[5]
*SlHAK20* Na^+^/K^+^ transporter	*Solanum pimpinellifolium* TS-21 and TS-422	*Solanum lycopersicum* TS-577 and TS-670	Salinity	6 bp indel in the coding sequence	[4]
*PaMBF1c* Multiprotein Bridging Factor 1c	*Polytrichastrum alpinum*	*Arabidopsis thaliana*	Multiple stresses (heat, salt, and ionic)	14 amino acid variations	[14]
*AnGolS1* Galactinol synthase1	*Ammopiptanthus nanus*	*Solanum lycopersicum*	Cold	Possible variations in the promoter and the coding sequence resulting in different gene inducibility and activity	[33]
*SsNHX1* Vacuolar Na^+^/H^+^ antiporter	*Suaeda salsa*	*Arabidopsis thaliana*	Cold Salinity	Possible variations in the coding sequence	[34]
*SbSOS1* Plasma membrane Na^+^/H^+^ antiporter	*Salicornia brachiata*	*Nicotiana tabacum*	Salinity	Possible variations in the coding sequence	[35]
*McHKT2 (McHKT1;2)* High-affinity K^+^ transporter	*Mesembryanthemum crystallinum*	*Arabidopsis thaliana*	Salinity	Variations in the coding sequence resulting in 13 contiguous threonine residues in *McHKT2*	[36]
*TsHKT1;2* High-affinity K^+^ transporter	*Thellungiella salsuginea*	*Arabidopsis thaliana* *Solanum lycopersicum*	Salinity	Variations in the coding sequence resulting in amino acid variation in the second pore loop	[37,38] [39]

## References

[1] Vasil I.K. (2008). A history of plant biotechnology: From the Cell Theory of Schleiden and Schwann to biotech crops. Plant Cell Rep..

[2] Zhang H., Li Y., Zhu J.K. (2018). Developing naturally stress-resistant crops for a sustainable agriculture. Nat. Plants.

[3] Wang X.L., Wang H.W., Liu S.X., Ferjani A., Li J.S., Yan J.B., Yang X.H., Qin F. (2016). Genetic variation in ZmVPP1 contributes to drought tolerance in maize seedlings. Nat. Genet..

[4] Wang Z., Hong Y., Zhu G., Li Y., Niu Q., Yao J., Hua K., Bai J., Zhu Y., Shi H. (2020). Loss of salt tolerance during tomato domestication conferred by variation in a Na^+^/K^+^ transporter. EMBO J..

[5] Hu Y., Mesihovic A., Jiménez-Gómez J.M., Röth S., Gebhardt P., Bublak D., Bovy A., Scharf K.-D., Schleiff E., Fragkostefanakis S. (2020). Natural variation in HsfA2 pre-mRNA splicing is associated with changes in thermotolerance during tomato domestication. New Phytol..

[6] Gilliham M., Able J.A., Roy S.J. (2017). Translating knowledge about abiotic stress tolerance to breeding programmes. Plant J..

[7] Boyer J.S. (1982). Plant Productivity and Environment. Science.

[8] Dwivedi S.L., Ceccarelli S., Blair M.W., Upadhyaya H.D., Are A.K., Ortiz R. (2016). Lancrace Germplasm for Improving Yield and Abiotic Stress Adaptation. Trends Plant Sci.

[9] Alavilli H., Lee H., Park M., Yun D.J., Lee B.H. (2018). Enhanced multiple stress tolerance in Arabidopsis by overexpression of the polar moss peptidyl prolyl isomerase FKBP12 gene. Plant Cell Rep..

[10] Li Z.Q., Li J.X., Li H.J., Shi Z.H., Zhang G.F. (2015). Overexpression of TsApx1 from Thellungiella salsuginea improves abiotic stress tolerance in transgenic Arabidopsis thaliana. Biol. Plant.

[11] Zhang Z.Y., Li J.J., Pan Y.H., Li J.L., Zhou L., Shi H.L., Zeng Y.W., Guo H.F., Yang S.M., Zheng W.W. (2017). Natural variation in CTB4a enhances rice adaptation to cold habitats. Nat. Commun.

[12] Xiong H., Yu J., Miao J., Li J., Zhang H., Wang X., Liu P., Zhao Y., Jiang C., Yin Z. (2018). Natural Variation in OsLG3 Increases Drought Tolerance in Rice by Inducing ROS Scavenging. Plant Physiol..

[13] Mao D.H., Xin Y.Y., Tan Y.J., Hu X.J., Bai J.J., Liu Z.Y., Yu Y.L., Li L.Y., Peng C., Fan T. (2019). Natural variation in the HAN1 gene confers chilling tolerance in rice and allowed adaptation to a temperate climate. Proc. Natl. Acad. Sci. USA.

[14] Alavilli H., Lee H., Park M., Lee B.H. (2017). Antarctic Moss Multiprotein Bridging Factor 1c Overexpression in Arabidopsis Resulted in Enhanced Tolerance to Salt Stress. Front. Plant Sci.

[15] Ren Z.H., Zheng Z.M., Chinnusamy V., Zhu J.H., Cui X.P., Iida K., Zhu J.K. (2010). RAS1, a quantitative trait locus for salt tolerance and ABA sensitivity in Arabidopsis. Proc. Natl. Acad. Sci. USA.

[16] Julkowska M.M., Klei K., Fokkens L., Haring M.A., Schranz M.E., Testerink C. (2016). Natural variation in rosette size under salt stress conditions corresponds to developmental differences between Arabidopsis accessions and allelic variation in the LRR-KISS gene. J. Exp. Bot..

[17] Ma Y., Dai X., Xu Y., Luo W., Zheng X., Zeng D., Pan Y., Lin X., Liu H., Zhang D. (2015). COLD1 confers chilling tolerance in rice. Cell.

[18] Liu C.T., Ou S.J., Mao B.G., Tang J.Y., Wang W., Wang H.R., Cao S.Y., Schlappi M.R., Zhao B.R., Xiao G.Y. (2018). Early selection of bZIP73 facilitated adaptation of japonica rice to cold climates. Nat. Commun.

[19] Lu G., Wu F.Q., Wu W., Wang H.J., Zheng X.M., Zhang Y., Chen X., Zhou K., Jin M., Cheng Z. (2014). Rice LTG1 is involved in adaptive growth and fitness under low ambient temperature. Plant J..

[20] Fujino K., Sekiguchi H., Matsuda Y., Sugimoto K., Ono K., Yano M. (2008). Molecular identification of a major quantitative trait locus, qLTG3–1, controlling low-temperature germinability in rice. Proc. Natl. Acad. Sci. USA.

[21] Saito K., Hayano-Saito Y., Kuroki M., Sato Y. (2010). Map-based cloning of the rice cold tolerance gene Ctb1. Plant Sci..

[22] Liu F., Xu W., Song Q., Tan L., Liu J., Zhu Z., Fu Y., Su Z., Sun C. (2013). Microarray-assisted fine-mapping of quantitative trait loci for cold tolerance in rice. Mol. Plant.

[23] Zhao J., Zhang S., Dong J., Yang T., Mao X., Liu Q., Wang X., Liu B. (2017). A novel functional gene associated with cold tolerance at the seedling stage in rice. Plant Biotechnol. J..

[24] Xiao N., Gao Y., Qian H., Gao Q., Wu Y., Zhang D., Zhang X., Yu L., Li Y., Pan C. (2018). Identification of Genes Related to Cold Tolerance and a Functional Allele That Confers Cold Tolerance. Plant Physiol..

[25] Li X.M., Chao D.Y., Wu Y., Huang X.H., Chen K., Cui L.G., Su L., Ye W.W., Chen H., Chen H.C. (2015). Natural alleles of a proteasome alpha 2 subunit gene contribute to thermotolerance and adaptation of African rice. Nat. Genet..

[26] Singh B.P., Jayaswal P.K., Singh B., Singh P.K., Kumar V., Mishra S., Singh N., Panda K., Singh N.K. (2015). Natural allelic diversity in OsDREB1F gene in the Indian wild rice germplasm led to ascertain its association with drought tolerance. Plant Cell Rep..

[27] Ye N.H., Wang F.Z., Shi L., Chen M.X., Cao Y.Y., Zhu F.Y., Wu Y.Z., Xie L.J., Liu T.Y., Su Z.Z. (2018). Natural variation in the promoter of rice calcineurin B-like protein10 (OsCBL10) affects flooding tolerance during seed germination among rice subspecies. Plant J..

[28] Liu S.X., Wang X.L., Wang H.W., Xin H.B., Yang X.H., Yan J.B., Li J.S., Tran L.S.P., Shinozaki K., Yamaguchi-Shinozaki K. (2013). Genome-Wide Analysis of ZmDREB Genes and Their Association with Natural Variation in Drought Tolerance at Seedling Stage of Zea mays L.. PLoS Genet..

[29] Mao H., Wang H., Liu S., Li Z., Yang X., Yan J., Li J., Tran L.S., Qin F. (2015). A transposable element in a NAC gene is associated with drought tolerance in maize seedlings. Nat. Commun.

[30] Zhang X.M., Mi Y., Mao H.D., Liu S.X., Chen L.M., Qin F. (2020). Genetic variation in ZmTIP1 contributes to root hair elongation and drought tolerance in maize. Plant Biotechnol. J..

[31] Munns R., James R.A., Xu B., Athman A., Conn S.J., Jordans C., Byrt C.S., Hare R.A., Tyerman S.D., Tester M. (2012). Wheat grain yield on saline soils is improved by an ancestral Na(+) transporter gene. Nat. Biotechnol..

[32] Bian X.H., Li W., Niu C.F., Wei W., Hu Y., Han J.Q., Lu X., Tao J.J., Jin M., Qin H. (2020). A class B heat shock factor selected for during soybean domestication contributes to salt tolerance by promoting flavonoid biosynthesis. New Phytol..

[33] Liu Y., Zhang L., Meng S., Liu Y., Zhao X., Pang C., Zhang H., Xu T., He Y., Qi M. (2020). Expression of galactinol synthase from Ammopiptanthus nanus in tomato improves tolerance to cold stress. J. Exp. Bot..

[34] Li J., Jiang G., Huang P., Ma J., Zhang F. (2007). Overexpression of the Na^+^/H^+^ antiporter gene from Suaeda salsa confers cold and salt tolerance to transgenic Arabidopsis thaliana. Plant CellTissue Organ. Cult..

[35] Yadav N.S., Shukla P.S., Jha A., Agarwal P.K., Jha B. (2012). The SbSOS1 gene from the extreme halophyte Salicornia brachiata enhances Na^+^ loading in xylem and confers salt tolerance in transgenic tobacco. BMC Plant Biol..

[36] Nishijima T., Furuhashi M., Sakaoka S., Morikami A., Tsukagoshi H. (2017). Ectopic expression of Mesembryanthemum crystallinum sodium transporter McHKT2 provides salt stress tolerance in Arabidopsis thaliana. Biosci. Biotechnol. Biochem..

[37] Ali Z., Park H.C., Ali A., Oh D.H., Aman R., Kropornicka A., Hong H., Choi W., Chung W.S., Kim W.Y. (2012). TsHKT1;2, a HKT1 homolog from the extremophile Arabidopsis relative Thellungiella salsuginea, shows K(+) specificity in the presence of NaCl. Plant Physiol..

[38] Ali A., Raddatz N., Aman R., Kim S., Park H.C., Jan M., Baek D., Khan I.U., Oh D.H., Lee S.Y. (2016). A Single Amino-Acid Substitution in the Sodium Transporter HKT1 Associated with Plant Salt Tolerance. Plant Physiol..

[39] Vu T.V., Sivankalyani V., Kim E.J., Doan D.T.H., Tran M.T., Kim J., Sung Y.W., Park M., Kang Y.J., Kim J.Y. (2020). Highly efficient homology-directed repair using CRISPR/Cpf1-geminiviral replicon in tomato. Plant Biotechnol. J..

[40] Meyer R.S., DuVal A.E., Jensen H.R. (2012). Patterns and processes in crop domestication: An historical review and quantitative analysis of 203 global food crops. New Phytol..

[41] Purugganan M.D., Fuller D.Q. (2009). The nature of selection during plant domestication. Nature.

[42] Lakhanpaul S., Singh V., Kumar S., Bhardwaj D., Bhat K.V., Tuteja N., Gill S.S., Tiburcio A.F., Tuteja R. (2012). Sesame: Overcoming the Abiotic Stresses in the Queen of Oilseed Crops. Improving Crop Resistance to Abiotic Stress.

[43] James R.A., Blake C., Zwart A.B., Hare R.A., Rathjen A.J., Munns R. (2012). Impact of ancestral wheat sodium exclusion genes Nax1 and Nax2 on grain yield of durum wheat on saline soils. Funct. Plant Biol..

[44] Horie T., Hauser F., Schroeder J.I. (2009). HKT transporter-mediated salinity resistance mechanisms in Arabidopsis and monocot crop plants. Trends Plant Sci..

[45] Munns R., Tester M. (2008). Mechanisms of salinity tolerance. Annu. Rev. Plant Biol..

[46] Schachtman D.P., Schroeder J.I. (1994). Structure and transport mechanism of a high-affinity potassium uptake transporter from higher plants. Nature.

[47] Waters S., Gilliham M., Hrmova M. (2013). Plant High-Affinity Potassium (HKT) Transporters involved in salinity tolerance: Structural insights to probe differences in ion selectivity. Int. J. Mol. Sci..

[48] Katori T., Ikeda A., Iuchi S., Kobayashi M., Shinozaki K., Maehashi K., Sakata Y., Tanaka S., Taji T. (2010). Dissecting the genetic control of natural variation in salt tolerance of Arabidopsis thaliana accessions. J. Exp. Bot..

[49] Imai H., Noda Y., Tamaoki M. (2015). Alteration of Arabidopsis SLAC1 promoter and its association with natural variation in drought tolerance. Plant Signal. Behav..

[50] Jha D., Shirley N., Tester M., Roy S.J. (2010). Variation in salinity tolerance and shoot sodium accumulation in Arabidopsis ecotypes linked to differences in the natural expression levels of transporters involved in sodium transport. Plant Cell Environ..

[51] Wang Y.P., Yang L., Zheng Z.M., Grumet R., Loescher W., Zhu J.K., Yang P.F., Hu Y.L., Chan Z.L. (2013). Transcriptomic and Physiological Variations of Three Arabidopsis Ecotypes in Response to Salt Stress. PLoS ONE.

[52] Bouchabke O., Chang F.Q., Simon M., Voisin R., Pelletier G., Durand-Tardif M. (2008). Natural Variation in Arabidopsis thaliana as a Tool for Highlighting Differential Drought Responses. PLoS ONE.

[53] Sharma S., Lin W.D., Villamor J.G., Verslues P.E. (2013). Divergent low water potential response in Arabidopsis thaliana accessions Landsberg erecta and Shahdara. Plant Cell Environ..

[54] Kesari R., Lasky J.R., Villamor J.G., Marais D.L.D., Chen Y.J.C., Liu T.W., Lin W., Juenger T.E., Verslues P.E. (2012). Intron-mediated alternative splicing of Arabidopsis P5CS1 and its association with natural variation in proline and climate adaptation. Proc. Natl. Acad. Sci. USA.

[55] Kovach M.J., Sweeney M.T., McCouch S.R. (2007). New insights into the history of rice domestication. Trends Genet..

[56] Sang T., Ge S. (2007). Genetics and phylogenetics of rice domestication. Curr. Opin. Genet. Dev..

[57] Lu B.-R., Cai X., Xin J. (2009). Efficient indica and japonica rice identification based on the InDel molecular method: Its implication in rice breeding and evolutionary research. Prog. Nat. Sci..

[58] E Z.G., Zhang Y.P., Zhou J.H., Wang L. (2014). Mini review roles of the bZIP gene family in rice. Genet. Mol. Res..

[59] Nijhawan A., Jain M., Tyagi A.K., Khurana J.P. (2008). Genomic survey and gene expression analysis of the basic leucine zipper transcription factor family in rice. Plant Physiol..

[60] Vaughan D.A., Lu B.-R., Tomooka N. (2008). The evolving story of rice evolution. Plant Sci..

[61] Menguer P.K., Sperotto R.A., Ricachenevsky F.K. (2017). A walk on the wild side: Oryza species as source for rice abiotic stress tolerance. Genet. Mol. Biol..

[62] Gupta P.C., O’Toole J.C. (1986). Upland Rice: A Global Perspective.

[63] Suzuki N., Sejima H., Tam R., Schlauch K., Mittler R. (2011). Identification of the MBF1 heat-response regulon of Arabidopsis thaliana. Plant J. Cell Mol. Biol..

[64] Suzuki N., Rizhsky L., Liang H., Shuman J., Shulaev V., Mittler R. (2005). Enhanced tolerance to environmental stress in transgenic plants expressing the transcriptional coactivator multiprotein bridging factor 1c. Plant Physiol..

[65] Qin D., Wang F., Geng X., Zhang L., Yao Y., Ni Z., Peng H., Sun Q. (2015). Overexpression of heat stress-responsive TaMBF1c, a wheat (*Triticum aestivum* L.) Multiprotein Bridging Factor, confers heat tolerance in both yeast and rice. Plant Mol. Biol..

[66] Cheng S. (1959). Ammopiptanthus Cheng f. A new genus of Leguminosae from central Asia. J. Bot. Ussr.

[67] Ge X.-J., Yu Y., Yuan Y.-M., Huang H.-W., Yan C. (2005). Genetic diversity and geographic differentiation in endangered Ammopiptanthus (*Leguminosae*) populations in desert regions of northwest China as revealed by ISSR analysis. Ann. Bot..

[68] Liu Y.D., Zhang L., Chen L.J., Ma H., Ruan Y.U., Xu T., Xu C.Q., He Y., Qi M.F. (2016). Molecular cloning and expression of an encoding galactinol synthase gene (AnGolS1) in seedling of Ammopiptanthus nanus. Sci. Rep.-UK.

[69] Selvaraj M.G., Ishizaki T., Valencia M., Ogawa S., Dedicova B., Ogata T., Yoshiwara K., Maruyama K., Kusano M., Saito K. (2017). Overexpression of an Arabidopsis thaliana galactinol synthase gene improves drought tolerance in transgenic rice and increased grain yield in the field. Plant Biotechnol. J..

[70] Apse M.P., Aharon G.S., Snedden W.A., Blumwald E. (1999). Salt Tolerance Conferred by Overexpression of a Vacuolar Na+/H+ Antiport in Arabidopsis. Science.

[71] Shi H., Lee B.H., Wu S.J., Zhu J.K. (2003). Overexpression of a plasma membrane Na+/H+ antiporter gene improves salt tolerance in Arabidopsis thaliana. Nat. Biotechnol..

[72] Pehlivan N., Sun L., Jarrett P., Yang X., Mishra N., Chen L., Kadioglu A., Shen G., Zhang H. (2016). Co-overexpressing a Plasma Membrane and a Vacuolar Membrane Sodium/Proton Antiporter Significantly Improves Salt Tolerance in Transgenic Arabidopsis Plants. Plant Cell Physiol..

[73] Bohnert H.J., Cushman J.C. (2000). The Ice Plant Cometh: Lessons in Abiotic Stress Tolerance. J. Plant Growth Regul..

[74] Ali A., Khan I.U., Jan M., Khan H.A., Hussain S., Nisar M., Chung W.S., Yun D.J. (2018). The High-Affinity Potassium Transporter EpHKT1;2 From the Extremophile Eutrema parvula Mediates Salt Tolerance. Front. Plant Sci..

[75] Møller I.S., Gilliham M., Jha D., Mayo G.M., Roy S.J., Coates J.C., Haseloff J., Tester M. (2009). Shoot Na^+^ exclusion and increased salinity tolerance engineered by cell type-specific alteration of Na^+^ transport in Arabidopsis. Plant Cell.

[76] Chen H., Chen X., Gu H., Wu B., Zhang H., Yuan X., Cui X. (2014). GmHKT1;4, a novel soybean gene regulating Na^+^/K^+^ ratio in roots enhances salt tolerance in transgenic plants. Plant Growth Regul..

[77] Chen H., He H., Yu D. (2011). Overexpression of a novel soybean gene modulating Na^+^ and K^+^ transport enhances salt tolerance in transgenic tobacco plants. Physiol. Plant.

[78] Ren Z., Liu Y., Kang D., Fan K., Wang C., Wang G., Liu Y. (2015). Two alternative splicing variants of maize HKT1;1 confer salt tolerance in transgenic tobacco plants. Plant CellTissue Organ. Cult. (Pctoc.).

[79] Ali A., Cheol Park H., Aman R., Ali Z., Yun D.J. (2013). Role of HKT1 in Thellungiella salsuginea, a model extremophile plant. Plant Signal. Behav..

[80] Vinocur B., Altman A. (2005). Recent advances in engineering plant tolerance to abiotic stress: Achievements and limitations. Curr. Opin. Biotechnol..

[81] Wang W., Vinocur B., Altman A. (2003). Plant responses to drought, salinity and extreme temperatures: Towards genetic engineering for stress tolerance. Planta.

